# Widely conserved AHL transcription factors are essential for *NCR* gene expression and nodule development in *Medicago*

**DOI:** 10.1038/s41477-022-01326-4

**Published:** 2023-01-09

**Authors:** Senlei Zhang, Ting Wang, Rui M. Lima, Aladár Pettkó-Szandtner, Attila Kereszt, J. Allan Downie, Eva Kondorosi

**Affiliations:** 1 Institute of Plant Biology, Biological Research Centre, Szeged, Hungary; 2 Laboratory of Proteomic Research, Biological Research Centre, Szeged, Hungary; 3 John Innes Centre, Norwich, UK

**Keywords:** Rhizobial symbiosis, Plant molecular biology

## Abstract

Symbiotic nitrogen fixation by *Rhizobium* bacteria in the cells of legume root nodules alleviates the need for nitrogen fertilizers. Nitrogen fixation requires the endosymbionts to differentiate into bacteroids which can be reversible or terminal. The latter is controlled by the plant, it is more beneficial and has evolved in multiple clades of the Leguminosae family. The plant effectors of terminal differentiation in inverted repeat-lacking clade legumes (IRLC) are nodule-specific cysteine-rich (NCR) peptides, which are absent in legumes such as soybean where there is no terminal differentiation of rhizobia. It was assumed that *NCR*s co-evolved with specific transcription factors, but our work demonstrates that expression of *NCR* genes does not require *NCR*-specific transcription factors. Introduction of the *Medicago truncatula NCR169* gene under its own promoter into soybean roots resulted in its nodule-specific expression, leading to bacteroid changes associated with terminal differentiation. We identified two AT-Hook Motif Nuclear Localized (AHL) transcription factors from both *M. truncatula* and soybean nodules that bound to AT-rich sequences in the *NCR169* promoter inducing its expression. Whereas mutation of *NCR169* arrested bacteroid development at a late stage, the absence of MtAHL1 or MtAHL2 completely blocked bacteroid differentiation indicating that they also regulate other *NCR* genes required for the development of nitrogen-fixing nodules. Regulation of *NCR*s by orthologous transcription factors in non-IRLC legumes opens up the possibility of increasing the efficiency of nitrogen fixation in legumes lacking *NCR*s.

## Abstract (web summary)

Expression of *Medicago truncatula* symbiosis-specific nodule-specific cysteine-rich (NCR) genes in soybean—which lacks NCR—is induced by conserved transcription factors in the symbiotic nodule cells and provokes features of terminal differentiation of endosymbiotic *Rhizobium* bacteroids.

## Main

Legume plants limited for nitrogen establish an intracellular symbiosis with bacteria called rhizobia, resulting in the formation of nitrogen-fixing root nodules. Nodules can be of indeterminate or determinate types, depending on whether the nodule meristem is persistent or limited during development. In the indeterminate nodules, there is an age and differentiation gradient from the apex to the nodule base resulting in the formation of a meristematic zone at the apex (ZI), an invasion zone (ZII) where rhizobia infect nodule cells, an interzone (IZ) where endosymbiont differentiation starts and a nitrogen-fixing zone (ZIII) containing fully differentiated bacteroids. In determinate nodules, synchronized development of the newly formed cells takes place after meristematic activity ceases. Depending on the legume, bacteroid differentiation can be reversible or terminal and can occur in both nodule types^1^. In the nodules of soybean (*Glycine max*), *Leucaena* or the model legume *Lotus japonicus*, bacteroids are similar to free-living bacteria and they can regrow from nodules, so their fate is reversible. In the nodules of inverted repeat-lacking clade (IRLC) legumes, which include peas, clovers and medics, or of dalbergoid legumes such as peanut and *Aeschynomene*, bacteroid differentiation is associated with the loss of cell division capacity, genome amplification, increased cell size and altered membrane permeability^2,3^. This terminal bacteroid differentiation is provoked by plant-made nodule-specific cysteine-rich (NCR or NCR-like) peptides^4^ and is presumably more beneficial for the plants by providing more efficient nitrogen fixation^5,6^. *NCR* genes have evolved uniquely in IRLC legumes and are absent from other legumes and other plants.

In the model legume *Medicago truncatula*, ~700 genes encode secreted NCR peptides. The mature peptides are mostly 30–50 amino acids long, highly divergent and are characterized by four or six conserved cysteines^7^. The *NCR* genes are expressed exclusively in the symbiotic nodule cells in consecutive waves^8,9^. This results in the delivery of different sets of NCRs to bacteroids as they develop. The rhizobial *bacA* gene encoding a peptide transporter is required for NCR-induced bacteroid differentiation and symbiotic nitrogen fixation^10,11^, but *bacA* (or its orthologue, *bclA*) is not required in rhizobia infecting legumes that lack NCR peptides^12^.

The function of only a few NCR peptides has been elucidated. One of them, NCR169, conserved only in species of the closely related *Medicago* and *Melilotus* genera, is essential for full differentiation of bacteroids and the development of nitrogen-fixing nodules in *M. truncatula*^13^. *NCR169* is expressed in IZ and ZIII and its absence in the *M. truncatula dnf7-2* mutant provoked degradation of immature bacteroids and the absence of a nitrogen-fixing zone. Because the *NCR169* promoter shows conserved features found in many *NCR* genes^14^, *NCR169* was ideally suited for analysis of *NCR* gene regulation. The *dnf7-2* mutant was successfully complemented with *NCR169* with a 1,178 bp promoter region upstream of the translational start site^13^. In this work, we identified *cis*-acting elements and nodule-expressed DNA-binding proteins belonging to the Type I AT-Hook Motif Nuclear Localized (AHL) transcription factor family that are essential for the induction and proper expression of *NCR169* for the development of nitrogen-fixing root nodules. These AHLs are conserved in non-IRLC legumes and can induce expression of active NCR169 in symbiotic nodule cells.

## Results

### *NCR169* is expressed under its native promoter in soybean nodules

**Fig. 1:**
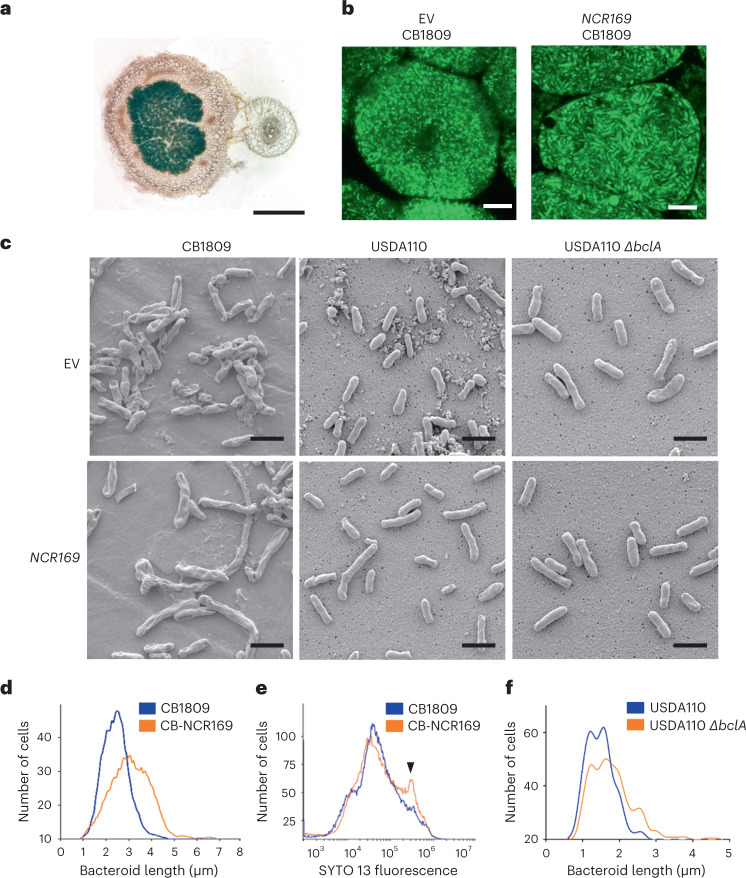
Analysis of the effects of the *M. truncatula NCR169* gene expression in soybean nodules. **a**, Expression of the 1,181 bp *NCR169* promoter::*GUS* reporter in soybean nodules detected with blue staining due to GUS enzyme activity. Scale bar, 1 mm. **b**, *B. japonicus* CB1809 bacteroids in SYTO 9/PI-stained sections of soybean nodules transformed with the empty vector (EV) or with *NCR169* visualized by confocal microscopy. **c**, SEM of wild-type (CB1809 and USDA110) and *ΔbclA* mutant *Bradyrhizobium* bacteroids isolated from empty vector (upper) and *NCR169*-transformed (lower) nodules. **d**, The lengths of 500 bacteroids isolated from control (blue trace) and *NCR169*-expressing (orange trace) nodules were measured with a Nano Measurer and plotted as frequency distributions. **e**, The DNA content of isolated and SYTO 13-stained bacteroids from the control (blue trace) and the *NCR169*-expressing (orange trace) nodules were measured by flow cytometry and plotted as frequency distributions. The subpopulation with increased DNA content is indicated by an arrow. **f**, The lengths of wild-type (blue trace) and *ΔbclA* mutant (orange trace) bacteroids isolated from *NCR169*-expressing nodules were measured and plotted as frequency distributions. Scale bar, 4 μm (confocal images) and 2 μm (SEM images). All experiments were repeated three times and similar results were obtained.

The 1,181 bp promoter region of *NCR169* known to be sufficient for normal expression in *M. truncatula*^13^ was fused with the *GUS* reporter gene and introduced into soybean roots by *Agrobacterium rhizogenes*-mediated root transformation. The nodules formed with *Bradyrhizobium japonicum* CB1809 were then stained for GUS activity. Unexpectedly, and detected by blue staining, the reporter gene was expressed in the symbiotic soybean nodule cells (Fig. 1a), revealing that transcription factors in soybean nodules can induce the promoter of the *M. truncatula NCR169* gene.

*NCR169* preceded by this 1,181 bp promoter region was used to transform soybean roots to test whether NCR169 affected soybean bacteroid morphology. Nodules induced by *Bradyrhizobium japonicum* CB1809 on soybean roots, transformed either with *NCR169* or with the empty vector, were sectioned and stained with SYTO 9 and propidium iodide (PI) to visualize living (green) and dead (red) bacteroids respectively, using confocal microscopy (Fig. 1b). Comparison of nodules revealed that NCR169 did not affect the viability but induced the elongation of bacteroids. This was confirmed by scanning electron microscopy (SEM) of bacteroids isolated from control and *NCR169*-expressing nodules (Fig. 1c). The average length of bacteroids in the nodules expressing *NCR169* increased by ~25% (Fig. 1d) and was associated with a small but noticeable increase in the DNA content measured by flow cytometry (Fig. 1e).

**Extended Data Fig. 1:**
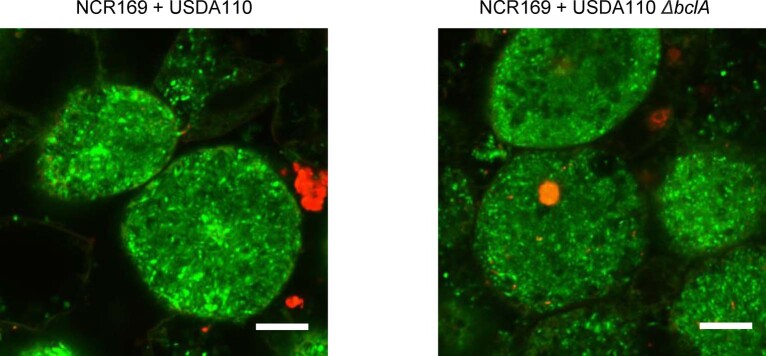
Viability of *B. japonicus* USDA110 and *B. japonicus* USDA110 *ΔbclA* bacteroids in soybean nodules expressing *NCR169* assessed by Live (SYTO9)/Dead (PI) staining. Plant nuclei are stained red by PI. Scale bars indicate 4 μm. Similar results were obtained in three independent experiments.

In *Sinorhizobium meliloti*, the symbiotic partner of *M. truncatula*, *bacA* is required for NCR-mediated differentiation of bacteroids and symbiotic nitrogen fixation in *M. truncatula*^11^. The homologous gene in *Bradyrhizobium* (*bclA*) is not required for nitrogen fixation in soybean nodules but is essential in *Aeschynomene* legumes producing NCR-like peptides governing terminal bacteroid differentiation^12,15,16^. To assess the requirement of BclA for NCR169-induced bacteroid elongation, soybean roots transformed either with *NCR169* or the vector control were inoculated with wild-type *B. japonicum* USDA110 or its *bclA* mutant. Live/dead staining revealed that both wild-type and *bclA* bacteroids were alive in transgenic nodules (Extended Data Fig. 1), but *NCR169*-induced cell elongation occurred only in nodules infected by wild-type *B. japonicum* and not in the case of the *bclA* mutant (Fig. 1c,f). This result supports the known interdependence of BclA/BacA and NCR functions. Moreover, the NCR169-induced increase in the size of soybean bacteroids is in line with the role of *NCR169* in *M. truncatula*, confirming that the NCR169 peptide is expressed in and can function in soybean nodules.

### Delimitation of the minimal *NCR169* promoter region

**Extended Data Fig. 2:**
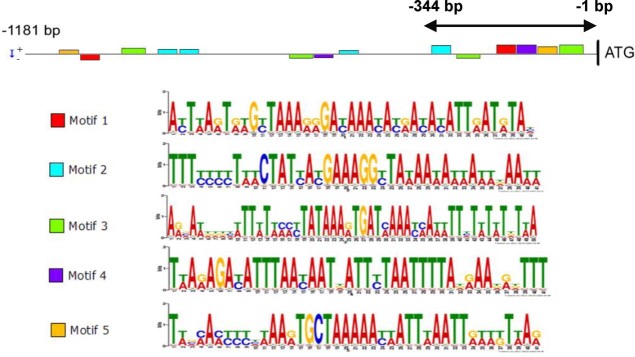
Occurrence of the five reported motifs in the 1181 bp promoter preceding the *NCR169* translation start. The analysis was conducted with MAST tool in the MEME Suite^39^ (https://meme-suite.org/meme/index.html). Sequences matched to the five motifs conserved in promoters of *NCR* genes as reported^14^ are highlighted. The heights of the rectangles represent the levels of similarity. The sequences of the conserved five motifs, are densely clustered in the upstream 344 bp region, which is indicated. The conservation of nucleotides in the sequences of the five motifs are shown by letter height.

Previous bioinformatic analysis of 209 *NCR* gene promoters from *M. truncatula* revealed five motifs in *NCR* promoters^14^. In the *NCR169* promoter, sequences with similarity to motifs 2, 1, 4, 5 and 3 were present in that order ~400 bp upstream of the translation start, with motif 3 closest to the translational start. However, potential motifs were also found further upstream (Extended Data Fig. 2).

**Fig. 2:**
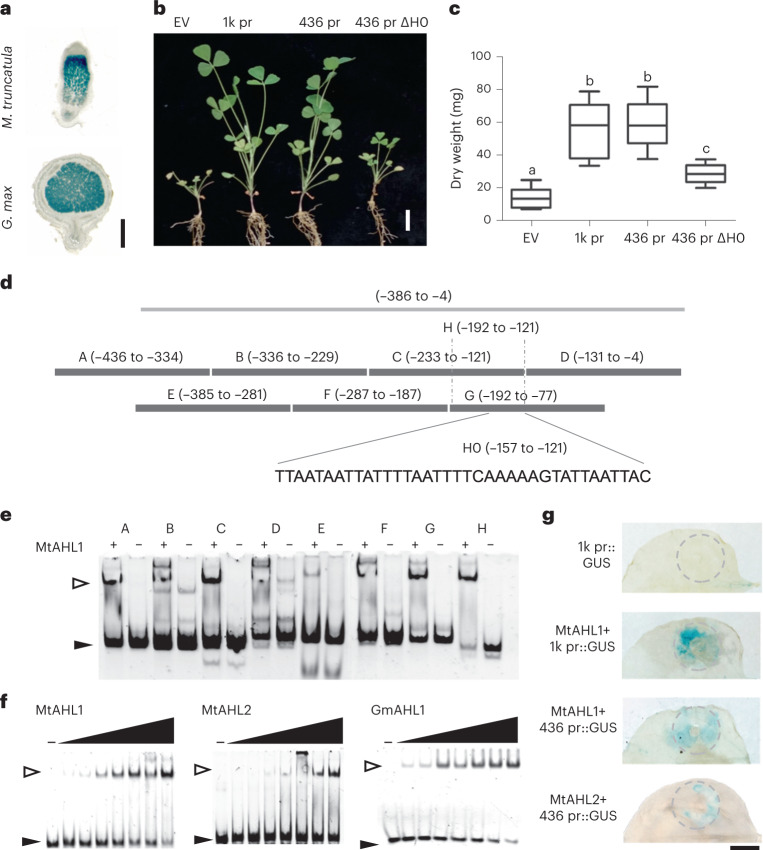
Identification of the *NCR169* promoter region essential for *NCR169* expression and for binding of MtAHLs. **a**, GUS enzyme activity (blue staining) induced by the 436 bp promoter region in nodules formed on hairy roots of *M. truncatula* and *G. max*. Scale bar, 1 mm. **b**, Complementation of the *NCR169* mutant *dnf7-2* with the *NCR169* coding sequence preceded by either the 1,181 bp (1k pr) or 436 bp promoter region (436 pr) or the 436 bp promoter region deleted for the H0 sequence (436 pr *Δ*H0). Scale bar, 1 cm. **c**, Average dry weights of plants shown in **b** were calculated (*n* = 10). In the boxplot, the central black line represents the median; the box limits are the upper and lower quartiles; the whiskers represent the lowest or highest data point within the 1.5 interquartile range of the lower or upper quartile. Different letters above the bars indicate significant differences (two-tailed unpaired *t* test, *P* < 0.0006). **d**, Locations of the DNA fragments used for the DNA affinity chromatography (grey line) and for the EMSA (black lines). **e**, Interactions of probes A–H with purified His-tagged MtAHL1 assessed with EMSA. **f**, Binding of purified MtAHL1, MtAHL2 and GmAHL1 at increasing concentrations to the H0 region. The black arrow indicates the free probe, and the hollow arrow indicates retarded bands. **g**, Induction of the 1,181 bp (1k pr::GUS) or 436 bp (436 pr::GUS) *NCR169* promoter::GUS by co-infiltration of *N. benthamiana* leaves with *A. tumefaciens* carrying *MtAHL1* or *MtAHL2*. The leaves were stained with X-Gluc for 72 h after infiltration. The dashed circle indicates the infiltrated area. Scale bar, 1 cm. Similar results were obtained in three independent repeats for all the experiments mentioned above.

To test whether the ~400 bp promoter region was sufficient for *NCR169* expression, 436 bp upstream of the translation start (including all five motifs) was fused with *GUS* and introduced into both *M*. *truncatula* and *G. max* roots using *A. rhizogenes*-mediated root transformation. The expression of this reporter fusion in both plants (Fig. 2a) was similar to that seen with the longer promoter (Fig. 1a). In *M. truncatula*, the spatial expression pattern of the 436 bp promoter::*GUS* was identical to that seen with the 1,181 bp promoter::*GUS*^13^ indicating that the 436 bp DNA fragment confers the correct, *NCR169*-specific expression pattern. In line with this, a construct containing the NCR169-coding region downstream of the 436 bp fragment also complemented *M. truncatula dnf7-2* (*ncr169* mutant) to a similar extent as the 1,181 bp promoter region, resulting in nitrogen fixation, normal plant development (Fig. 2b) and plant mass (Fig. 2c) under nitrogen limitation. This confirms that the 436 bp fragment is sufficient for proper production of NCR169.

### Identification of proteins interacting with *NCR169* promoter

First, the 1,181 bp promoter region was used as the bait target DNA in a yeast one-hybrid (Y1H) screen in which the prey proteins were from a *M. truncatula* nodule complementary DNA library expressing the proteins in fusion with the yeast GAL4 transcription activation domain (GAL4 AD)^17^. This screen resulted in the identification of seven putative DNA-binding proteins from *M. truncatula* nodules (Table 1). These were: an AT-Hook Motif DNA-binding family protein, MtAHL1; a basic helix–loop–helix domain class transcription factor; a MYB-like transcription factor family protein; a TCP family transcription factor; a transcription factor VOZ1-like protein; a BEL1-related homeotic protein; and a linker histone H1 and H5 family protein. Comparing the expression of these genes in different plant organs and in different nodule zones^8,18^ revealed that although each of them was expressed in both roots and nodules, only two, *MtAHL1* and the *BEL1*-related homeotic protein gene, were upregulated in nodules. Unlike *MtAHL1*, the *BEL1* transcripts were also detectable in leaves and petioles, whereas the transcripts of the other five genes were present in all plant organs.

**Table 1:** Interactors of *NCR169* promoter regions identified in Y1H library screens (1,181 bp) and DNA affinity chromatography (382 bp) from both *M. truncatula* and *G. max*

Interacting proteins	Gene ID	UniProt ID
**Proteins interacting with 1,181** **bp** ***NCR169*** **promoter in Y1H screening**		
MtAHL-1	Medtr4g098450	G7JGG2
Basic helix–loop–helix domain class transcription factor	Medtr4g081370	B7FHK4
MYB-like transcription factor	Medtr5g037080	G7K6Z9
TCP family transcription factor	Medtr7g028160	G7L283
Transcription factor VOZ1-like protein	Medtr4g088125	A0A072UMQ9
BEL1-related homeotic protein	Medtr8g098815	A0A072TUA6
Linker histone H1 and H5 family protein	Medtr6g079520	A0A072UBH0
***M. truncatula*** **proteins interacting with 382** **bp** ***NCR169*** **promoter in DNA pull-down assay**		
MtAHL1	Medtr4g098450	G7JGG2
MtAHL2	Medtr7g080980	G7KSI4
MtPHD1	Medtr4g015830	A0A072UGM4
MtPHD2	Medtr1g015185	A0A072VDQ0
Myb protein	Medtr7g020870	G7KSV7
Myb protein	Medtr7g103390	A0A072U4N3
MtPurα	Medtr4g083230	G7JHJ7
Zinc finger protein	Medtr1g053960	A0A072VIQ3
***G. max*** **proteins interacting with 382** **bp** ***NCR169*** **promoter in DNA pull-down assay**		
GmAHL1	Glyma.18G247200	C6TMY6
GmPHD1	Glyma.09G107000	C6T7X8
GmPurα	Glyma.07G225200	I1KMC5
SHOOT2 protein	Glyma.03G189600	C6TI90
Trihelix protein	Glyma.18G281800	I1N4Y2

**Extended Data Fig. 3:**
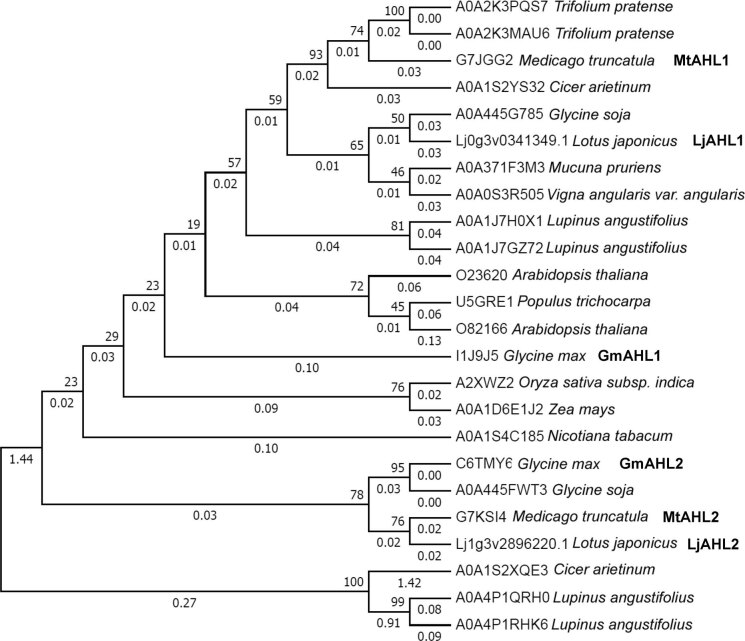
Phylogenetic tree of predicted AHL family proteins from selected plant species. Protein sequences were acquired from UniProt data base, except for those of *L. japonicus*, which were obtained from *Lotus* Base^40^. Protein sequences were aligned in MEGA 7^41^ with default settings and phylogenetic tree was generated with the aligned sequences using the neighbour-joining distance method. Stability of the branch topology in phylogenetic trees was tested using 1,000 bootstrap replicates. Bootstrap values are indicated. The *M. truncatula* (Mt), *G. max* (Gm) and *L. japonicus* (Lj) AHL1 and AHL2 proteins described in the text are indicated in bold.

As a complementary approach to identify potential *NCR169* transcription factors, DNA affinity chromatography pull-down experiments were carried out with a 382 bp DNA fragment that extended from −4 bp to −386 bp upstream of the translation start, encompassing all five potential promoter motifs described above. Nuclear protein extracts from *M. truncatula* and *G. max* nodules were added separately to this DNA fragment and proteins that bound were identified using mass spectrometry (MS). From the *M. truncatula* nuclear extracts, eight putative DNA-binding proteins were obtained: two of these were AT-Hook Motif nuclear proteins including MtAHL1 (as identified with the Y1H screen) and one we refer to as MtAHL2; two were plant homeodomain (PHD) finger alfin-like proteins (MtPHD1 and MtPHD2); two were Myb/SANT-like DNA-binding domain proteins; and the others were a Purα protein and a zinc finger C-x8-C-x5-C-x3-H type family protein (Table 1). With the *G. max* nuclear extracts, five putative DNA-binding proteins were identified (Table 1): based on phylogeny (Extended Data Fig. 3) one of these was GmAHL1, a probable orthologue of MtAHL1 (76% identity); one was a PHD finger alfin-like protein (GmPHD1), a probable orthologue of MtPHD1 (90% identity); one was a SHOOT2-like protein; one was a trihelix-like protein; and one was GmPurα, a probable homologue of MtPurα identified above (Table 1).

**Extended Data Fig. 4:**
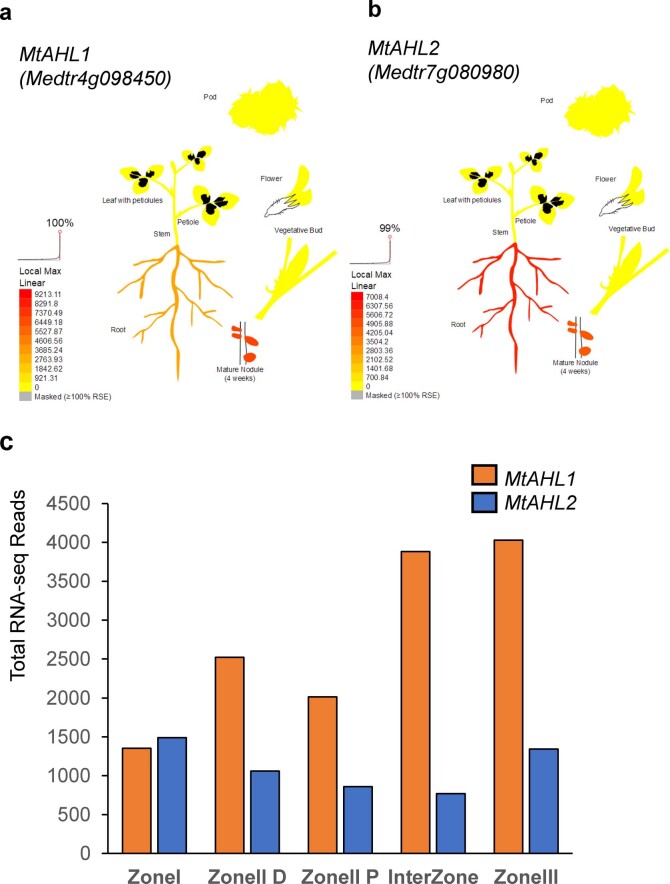
Diagram showing expression levels of *MtAHL1* and *MtAHL2* in different tissues and in different zones of the root nodule of *M. truncatula*. a, The expression level of *MtAHL1* (*Medtr4g098450*) is represented in different tissues by a color gradient, showing strong expression in nodules and lower expression in roots. b, The expression level of *MtAHL2* (*Medtr7g080980*) is stronger in roots and weaker in nodules. The images were generated using ePlant browser^42^ (http://bar.utoronto.ca/eplant_medicago/). The colour gradient is generated in ‘Local Max’ mode to discern expression pattern of the specific gene, rather than to indicate the actual expression level. c, The expression levels of *MtAHL1* and *MtAHL2* in the root nodule zones Zone I, Zone II distal (D) and proximal (P) regions, Inter Zone and Zone III as defined previously^18^.

**Extended Data Fig. 5:**
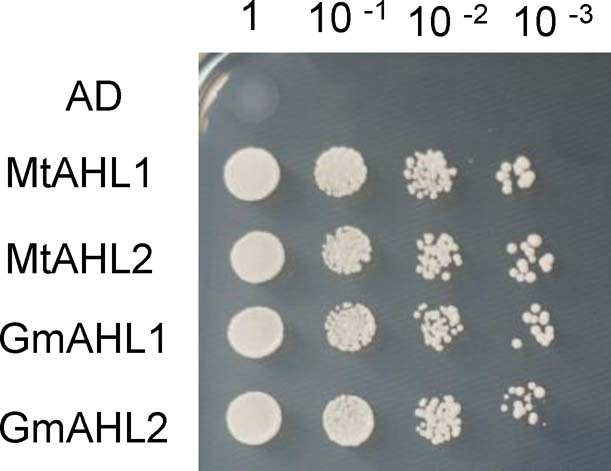
Detection of binding of the 1181 bp promoter region of *NCR169* to MtAHL1, MtAHL2, GmAHL1 or GmAHL2 in yeast one hybrid (YH1) assays. The yeast bait strain *S. cerevisiae* Y187 carrying pINT1-*NCR169 pr*-*HIS3* was transformed with plasmid pAD-GAL4 carrying *MtAHL1, MtAHL2, GmAHL1* or *GmAHL2*. The transformants were then diluted in 10-fold steps before cultivation on SD/Leu-His plates where growth indicates a positive interaction. The control strain carries the empty pAD-GAL4 vector (AD).

MtAHL1 was detected using the Y1H screen and by DNA affinity chromatography and its orthologue was identified by DNA affinity pull-down from *G. max*. Together with the nodule-specific expression pattern of *MtAHL1*, this suggested that MtAHL1 regulates *NCR169* expression. The DNA pull-down experiments using *M. truncatula* nodule nuclear extracts also identified MtAHL2, which is 75% identical to MtAHL1. The expression patterns of *MtAHL1* and *MtAHL2* were different, with *MtAHL2* showing higher expression in roots and lower expression in nodules than *MtAHL1* (Extended Data Fig. 4). Because the AHL family of regulators can act as heterotrimers^19^, we thought that MtAHL2 as well as its *G. max* orthologue, GmAHL2 (*Glyma.01G198800*), which we identified via phylogeny (Extended Data Fig. 3), could also be involved in *NCR169* regulation. The binding of MtAHL1, MtAHL2, GmAHL1 and GmAHL2 to the *NCR169* promoter was confirmed with Y1H assays (Extended Data Fig. 5). Probable orthologues of these AHL proteins from *M. truncatula* and *G. max* were also found in *L. japonicus* (Extended Data Fig. 3).

### MtAHLs bind to AT-rich sequences

The interaction between the minimal promoter region and the nodule-enhanced MtAHL1 protein was assayed using an electrophoretic mobility shift assay (EMSA) with purified MtAHL1 protein produced in *Escherichia coli* and seven overlapping DNA probes of ~100 bp (A–G) covering the 436 bp promoter region (Fig. 2d). MtAHL1 formed low-mobility complexes with the overlapping fragments C and G, but weaker complexes also formed with the other five fragments (Fig. 2e) indicating other possible binding sites.

The common region of fragments C and G (designated H, nucleotides −192 to −121) showed strong binding to MtAHL1 (Fig. 2e). Within fragment H there is an AT-rich region between nucleotides −157 and −121 that showed high similarity to the *NCR*-specific motif 4 (ref. 14). This 37 bp sequence (Fig. 2d), named H0, formed low-mobility complexes with MtAHL1, MtAHL2 and GmAHL1 (Fig. 2f). Analyses of RNA sequencing reads from public databases such as the Sequence Read Archive in the National Center for Biotechnology Information (https://www.ncbi.nlm.nih.gov/sra) revealed that transcription of *NCR169* starts 23 nucleotides upstream of the translation start site. The end of the H0 fragment is 98 bp from this deduced transcription start, a good location for a site of regulation. A further seven AT-rich regions with *P* values varying from 5.82 × 10^−5^ to 3.25 × 10^−15^ were identified on the 436 bp fragment and these could explain the secondary binding of MtAHL1 implied from the retardation of the other five DNA fragments (Fig. 2e).

The 436 bp *NCR169* promoter region fused to *GUS* was infiltrated into *N. benthamiana* leaves either alone or with *MtAHL1* or *MtAHL2* expressed from the ubiquitin promoter. Expression of GUS was observed only in the presence of *MtAHL1* or *MtAHL2* (Fig. 2g), confirming that either MtAHL1 or MtAHL2 can induce *NCR169*. The 37 bp H0 sequence between −157 bp and −121 bp was deleted from the 436 bp promoter region and the deleted promoter (436bpΔH0) was fused to the *NCR169* coding sequence. Unlike the intact 436 bp promoter, which was fully effective for complementing the *NCR169* deletion mutant *dnf7-2*, the absence of the H0 sequence resulted in poor complementation; plants were only slightly larger and greener than the *dnf7-2* mutant transformed with the empty vector (Fig. 2b,c).

### Both MtAHLs are crucial for normal nodule development

**Fig. 3:**
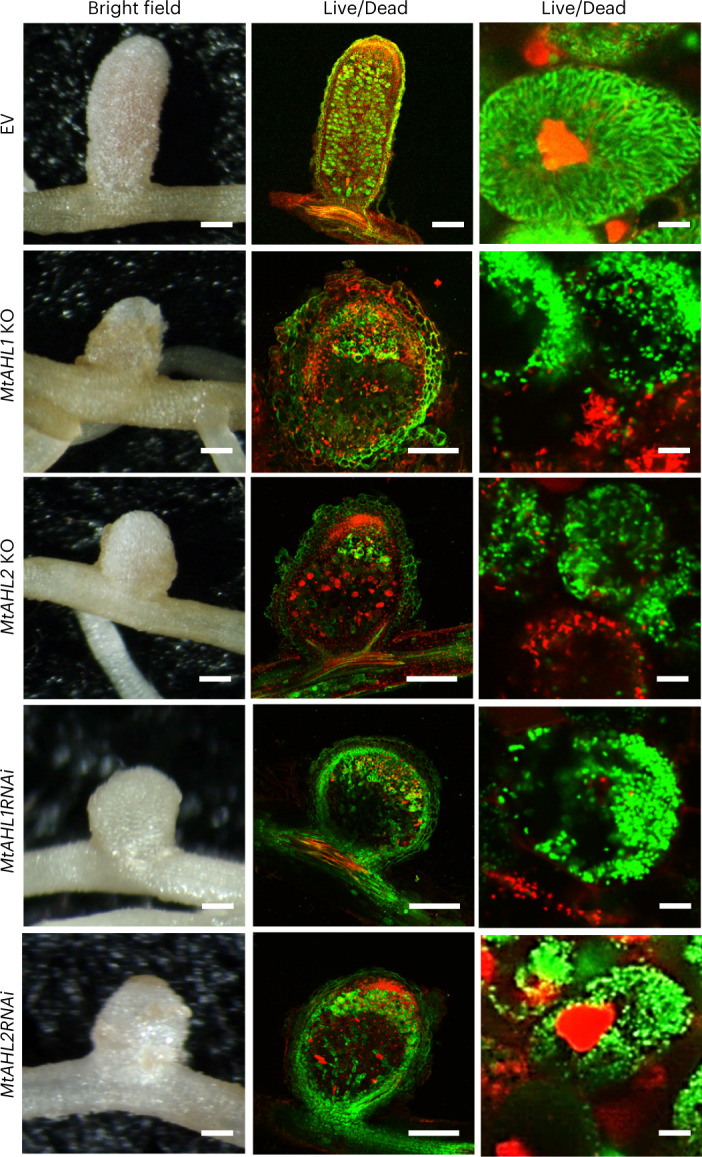
Assays of the effects of CRISPR–Cas9-mediated knockout mutations and RNAi-mediated gene silencing of *MtAHL1* or *MtAHL2* in *A. rhizogenes* transformed roots of *M. truncatula*. CRISPR–Cas9 knockout mutations were induced using the guide sequences described in Extended Data Fig. 6. RNAi was achieved by expressing hairpin constructs specifically targeting the *MtAHL1* or *MtAHL2* genes. Sections of transformed root nodules were examined by confocal microscopy after live/dead staining using SYTO 9, which stains live bacteroids green, and PI, which stains dead bacteroids and the nuclei of plant cells red. Scale bar, 1 mm (whole nodule) and 4 μm (symbiotic cell; right).

**Extended Data Fig. 6:**
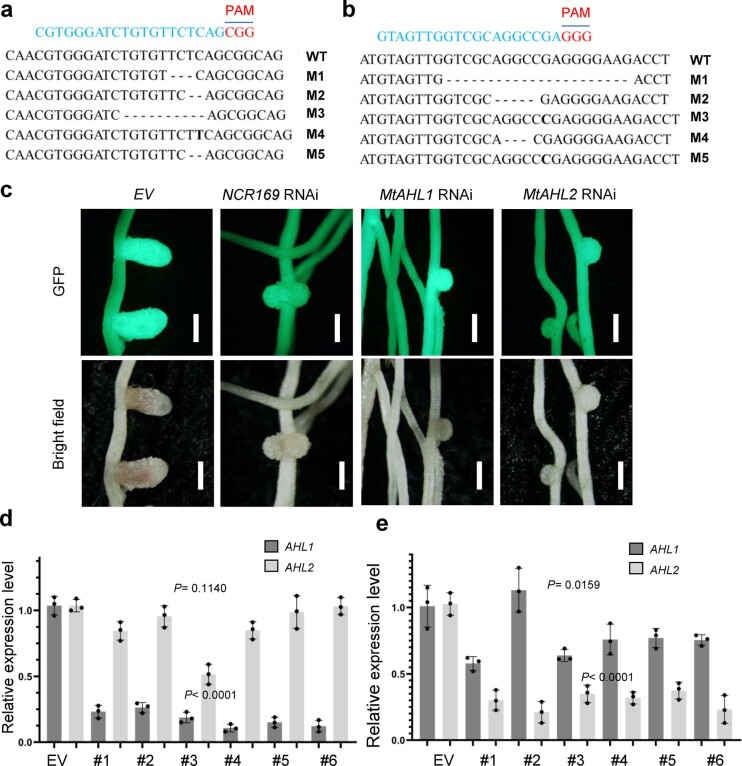
Analyses of the efficiency of inactivation and down-regulation of *MtAHL*s in hairy roots of *M. truncatul*a. a and b, CRISPR/Cas9 mediated mutations introduced into *MtAHL1* and *MtAHL2* were investigated using Illumina sequencing, typically with about 5000–10000 reads for each sampled nodules and representative modified sequences in individual nodules are shown. The data were analyzed on the CRISPResso2 website^43^ (https://crispresso.pinellolab.partners.org/). Representative sequences of mutated alleles of *MtAHL1* and *MtAHL2* are shown in a and b, respectively. The sgRNA sequences used are indicated in blue and the PAM site is in red. Nucleotide insertions are marked in bold. c, Photomicrographs of nodules formed on transgenic roots in which RNA interference was induced using RNAi constructs *MtAHL1* RNAi or *MtAHL2* RNAi; *NCR169* RNAi used as a phenotype control. The upper images captured GFP fluorescence and the lower images were taken with a bright field. Scale Bar indicates 2 mm. d and e, Expression levels of both *MtAHL1* and *MtAHL2* were measured by quantitative reverse transcription PCR in nodules formed on roots transformed with *MtAHL1* RNAi (d) or *MtAHL2* RNAi (e). Six roots were chosen for each analysis and experiments were repeated three times. Expression levels were normalized against the *Mt40S* ribosomal gene. The columns demonstrate the mean ± SE of fold change in gene expression in different roots compared with control. Dots, individual data points. *P* values are indicated for comparing expression level with the control (unpaired *t*-test was used).

The *MtAHL1* and *MtAHL2* genes were mutated in transformed roots of *M. truncatula* using CRISPR–Cas9 genome editing. Genomic modifications in the transgenic nodules were detected by analysing 5,000–10,000 reads of targeted amplicon sequencing that revealed deletions and/or insertions in the *MtAHL1* and *MtAHL2* genes. However, there were large variations in the efficacy of mutagenesis in the different transformed roots. Each nodule contained some wild-type DNA sequences, and the mutant sequence reads varied from 12.4% to 93.4% and from 25.7% to 98.8% in genome-edited *MtAHL1* and *MtAHL2* respectively (Extended Data Fig. 6a,b). Despite the apparent mosaic nature of these nodules that appear to contain wild-type and knockout mutant cells, only small, white non-fixing nodules developed on the *MtAHL1* and *MtAHL2* mutant lines (Fig. 3). SYTO 9 (live)/PI (dead) staining of nodule sections revealed successful infection of young nodule cells in ZII, but blocked bacteroid differentiation. Therefore, IZ and ZIII were absent and the nodule cells contained only dead non-enlarged bacteria. Loss of bacteroid viability was also observed in *M. truncatula dnf7-2* nodules^13^, but there the dead bacteroids were almost fully elongated.

Because the CRISPR–Cas9 system did not provide 100% knockout mutant nodules, expression of both *MtAHL1* and *MtAHL2* was downregulated with RNA interference (RNAi) using *A. rhizogenes*-mediated root transformation. This resulted in ~70%–90% downregulation of *MtAHL1* and 65%–80% downregulation of *MtAHL2*, as measured using a quantitative polymerase chain reaction with reverse transcription in the transgenic roots (Extended Data Fig. 6d,e). Nodules that developed on the RNAi lines were small, white, non-fixing, had the same nodule structure as the CRISPR–Cas9 mutant nodules and were devoid of differentiated live bacteroids (Fig. 3 and Extended Data Fig. 6c).

**Extended Data Fig. 7:**
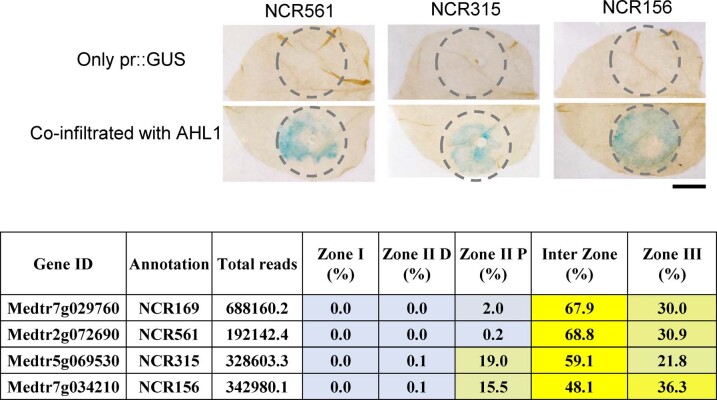
MtAHL1 activates the expression both ZII- and IZ-induced *NCR* genes. Approximately 500 bp of the promoters of selected *NCR* genes expressing at the same time (*NCR561*) as NCR169 or earlier (*NCR315, NCR165*) were cloned in front of the GUS coding sequence. The reporter constructs were infiltrated into *N. benthamiana* leaves either alone or co-infiltrated with MtAHL1. The leaves were stained with X-Gluc for 72 h after infiltration. Dashed circle indicates the infiltrated area. Scale bar indicates 1 cm.

Mutation or downregulation of *MtAHL*s in *M. truncatula* blocked bacteroid development at an earlier stage than observed in *dnf7-2* nodules lacking *NCR169* (ref. 13). This could be due to a lack of induction of both *NCR169* and other *NCR* genes. We searched for the motif 4 overlapping H0 in 1 kb regions upstream of *M. truncatula* genes using FIMO in the MEME suite (https://meme-suite.org/meme/tools/fimo). Of 292 genes with a *q* value below 0.01 (Supplementary Table 1), 280 encode NCR peptides and, with a few exceptions, are highly expressed in the proximal part of ZII, IZ and ZIII of the nodules suggesting that nodule expression of these genes may also require MtAHLs. To test this hypothesis, we fused to the GUS reporter gene ~500 bp promoter fragments of selected *NCR* genes from this list; some had very similar (*NCR561*: IZ–ZIII) and others had earlier (*NCR315* and *NCR165*: ZII–IZ–ZIII) expression compared with *NCR169*. We then tested whether their expression is induced by co-infiltrated *MtAHL1* in *N. benthamiana* leaves (Extended Data Fig. 7). The observed GUS activity indicates that MtAHL1 also regulates other *NCR* genes including some induced in ZII. This observation explains the observed early arrest of bacteroid development in the absence of *MtAHL*s.

**Extended Data Fig. 8:**
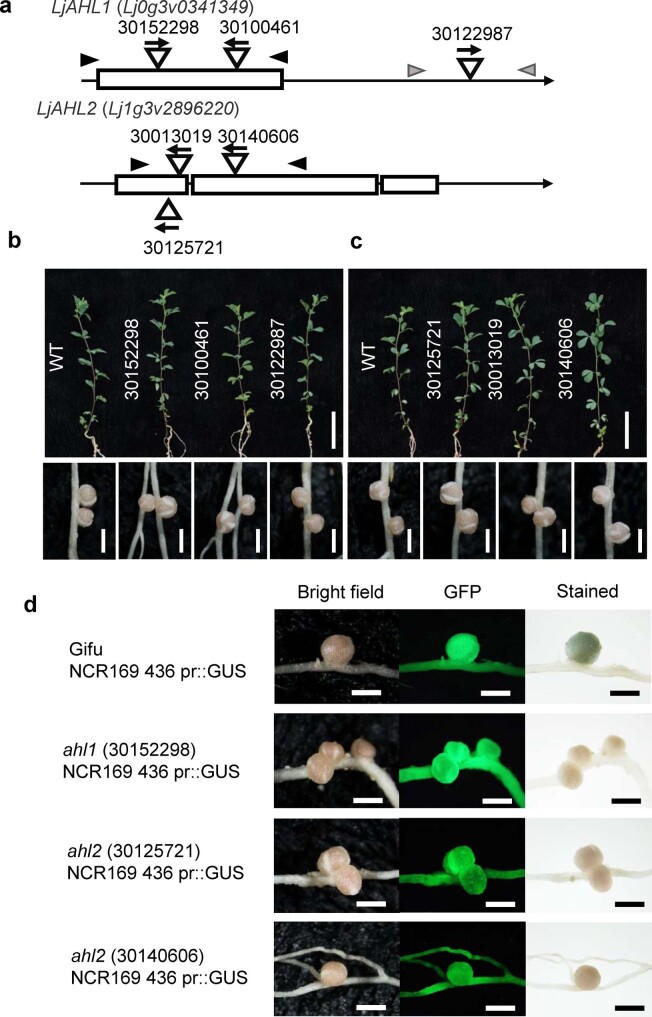
*L. japonicus AHL* mutants form effective nodules but fail to express the *pNCR169::GUS* reporter. *LjAHL1* and *LjAHL2* mutants were obtained from *Lotus* Base^44^ (https://lotus.au.dk/) and homozygous lines were selected with a PCR-based approach. a, The locations and orientations (arrows) of the *LORE1* insertions in *LjAHL1* (*Lj0g3v0341349)* and in *LjAHL2* (*Lj1g3v2896220)* are indicated. The open boxes represent exons and the pairs of primers used for genotyping to identify the homozygous mutants are indicated with arrowheads. Shoot growth and nodule morphology of the (b) *LjAHL1* and (c) *LjAHL2* mutant seedlings grown under N-limitation were indistinguishable from WT. Scale bars indicate 4 cm (plants) and 2 mm (nodules). d, The *LjAHL1* and *LjAHL2* mutants were transformed with the *GUS* gene driven by the 436 bp promoter of *NCR169*. Transgenic nodules were selected based on GFP fluorescence and stained for β-glucuronidase activity. Blue coloration was only observed in the nodules formed on the roots of the wild-type plants. Scale bar indicates 2 mm.

Non-NCR genes required for the establishment of symbiosis might also be regulated by AHL transcription factors. A way of assessing whether this is likely is to determine whether mutating *AHL* genes affects nitrogen-fixing symbiosis in a legume lacking NCR peptides. Because lines carrying mutations in *AHL1* or *AHL2* genes are available in *L. japonicus* (Extended Data Fig. 8a), we first confirmed that the 436 bp promoter of *NCR169* drives expression of the *GUS* reporter gene in *L. japonicus* nodules (Extended Data Fig. 8d). We then investigated whether homozygous *ahl1* or *ahl2* mutant lines can form an effective symbiosis and express the *pNCR169::GUS* fusion. All homozygous mutant lines formed normal pink nodules and the nodulated plants grew well in the absence of added nitrogen (Extended Data Fig. 8b,c). However, when we tried to express the *pNCR169::GUS* fusion in the mutants, no GUS activity could be detected in either mutant (Extended Data Fig. 8d). These results show that neither of the *L. japonicus AHL* genes regulates a gene required for nitrogen fixation, but expression of the *NCR169* gene in nodule tissues requires their concerted action. Moreover, this suggests that the symbiotic defects caused by mutations in *AHL* genes of *M. truncatula* are primarily due to effects on the regulation of *NCRs*.

## Discussion

Because *NCR* genes are found only in IRLC legumes, and their expression is developmentally regulated in nodules, we anticipated that they might be regulated by IRLC-specific transcription factors. However, we show that *M. truncatula NCR169* is induced in nodules of soybean and *L. japonicus* in which no *NCR* homologues could be found. Furthermore, the changes in soybean bacteroid size and DNA content induced by expression of *NCR169* are consistent with the NCR169 peptide being processed and delivered to the soybean bacteroids where it is taken up with the help of the BclA transporter. The initial acquisition of *NCR* genes in IRLC legumes involved recruitment of the existing nodule-specific protein secretory pathway across the plant-made symbiosome membrane to deliver the mature NCR peptides^4^. We can now infer that acquisition of *NCR* genes also involved recruitment of nodule-specific promoters regulated by existing transcription factors already present in nodules. A relatively short (436 bp) promoter upstream of the *NCR169* coding region is sufficient for nodule-specific expression of *NCR169* in all three legumes.

We identified two closely related transcription factors, MtAHL1 and MtAHL2, that bound to the previously recognized motif 4 in the *NCR169* promoter^14^. Within this motif, we identified a MtAHL1-binding site, which contains an AT-rich region of 37 bp (referred to as H0) required for normal *NCR169* expression. Deletion of this MtAHL-binding site severely affected complementation of the *NCR169*-defective *M. truncatula dnf7-2* mutant for symbiotic nitrogen fixation. This motif is conserved in the promotors of close to 300 *M. truncatula NCR* genes that are highly expressed in the proximal part of ZII, the IZ and ZIII of nodules (Supplementary Table 1).

The absence of either of the MtAHL1 and MtAHL2 transcription factors abolished the formation of nitrogen-fixing nodules in *M. truncatula* demonstrating their importance in symbiosis. Although essential for nodule induction of *NCR169*, they were also shown to induce other *NCR* genes. In the *dnf7-2* (*ncr169*) mutant nodules, bacteroid differentiation is arrested late, after nearly normal elongation and enlargement; these bacteroids are unable to fix nitrogen and are rapidly killed, resulting in the absence of nitrogen fixation ZIII^13^. However, in the absence or with low levels of MtAHL1 and MtAHL2, the nodule bacteria do not show any signs of bacteroid differentiation, are only viable in ZII and are already eliminated in the IZ (Fig. 3). This leads to early arrested growth of the nodules resulting in a spherical shape. These phenotypes and their ability to induce *NCR* genes of different expression patterns imply that MtAHL1 and MtAHL2 play crucial roles in regulation of other *NCR* genes required for full bacteroid differentiation and the development of fully functioning nitrogen-fixing nodules.

A role for AHL family proteins in nodule symbiosis has not been reported previously, although they have been recognized to be important for organ development in other plants. AHL family proteins contain one or two AT-hook(s) and a Plant and Prokaryote Conserved (PPC/DUF296) domain responsible for their interaction with themselves, other AHL proteins and non-AHL proteins^19^. They form homo- and heterotrimeric complexes and through their AT-hook domain(s) bind to DNA resulting in both the repression and induction of genes and biological pathways. They have been shown to be involved in axillary meristem maturation^20^, induction of somatic embryogenesis^21^, repression of hypocotyl elongation^22^, innate immunity^23^, patterning and differentiation of reproductive organs^24^, and affect the activity of various transposable element and transposable element-like repeat-containing genes such as the central floral repressor FLOWERING LOCUS C^25^. Binding of the AHLs to promoter elements rapidly changed histone H3 acetylation and methylation of the H3K9 residue via forming a complex with proteins participating in histone deacetylation^26^.

Given these roles in organ development, AHL family proteins may also be involved in the coordination of nodule development in legumes and the induction of nodule-specific genes such as *NCR* genes or possibly even their repression in other tissues. Of the more than 25 *AHL* genes of *M. truncatula*, at least 12 were expressed in nodules with variable patterns in the different nodule zones^18^. This might promote the formation of various AHL trimers that could differentially regulate different groups of genes. The lack of redundancy of *MtAHL1* and *MtAHL2* in *M. truncatula* nodules could be explained by each protein acting in different complexes or by the formation of different heterotrimeric complexes required for gene expression at different stages of nodule development. It seems likely that the symbiotic defects observed in the *MtAHL* knockdown lines and mutants were due to effects on regulation of both *NCR169* and some of the many other genes (primarily *NCR*s) induced in nodules^27^. If correct, this could explain why we were unable to distinguish different phenotypes after knocking down or mutating *MtAHL1* or *MtAHL2*.

**Extended Data Fig. 9:**
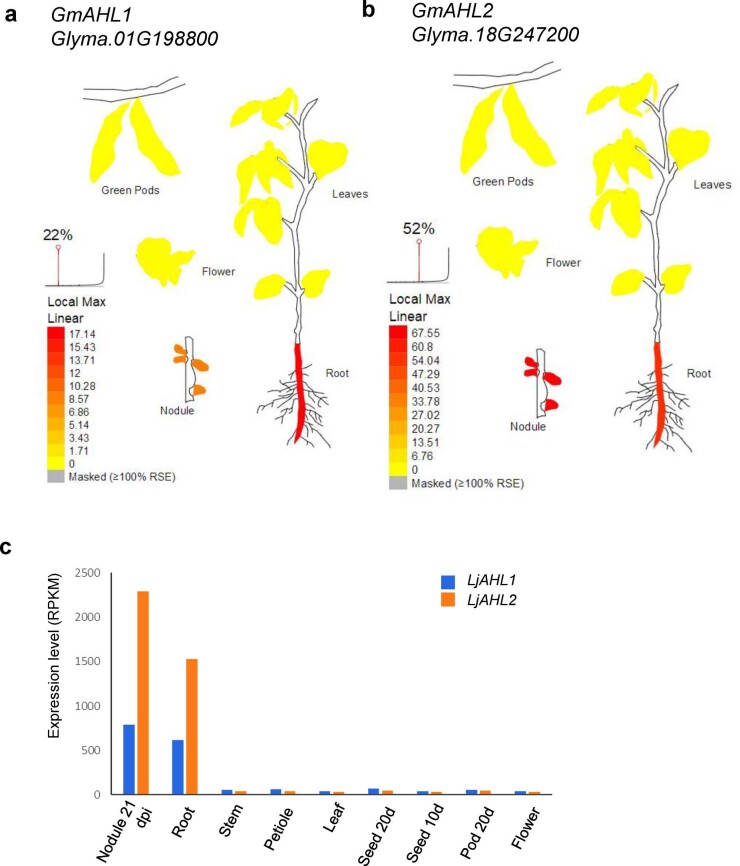
Patterns and levels of expression of *AHL1* and *AHL2* genes in soybean and *L. japonicus*. a, The expression level of *GmAHL1* (*Glyma.01G19800*) is represented in different tissues by a color gradient, showing strong expression in roots and lower expression in nodules. b, The expression level of *GmAHL2* (*Glyma.18G247200*) is strong in both nodules and roots. The images in a and b were generated using ePlant browser^42^ (http://bar.utoronto.ca/eplant_soybean/). The colour gradient is generated in ‘Local Max’ mode to discern expression pattern of the specific gene, rather than to indicate the actual expression level. c, The expression levels of *LjAHL1* (probe ID: Ljwgs_027513.1_at) and *LjAHL2* (probe ID: Ljwgs_062607.1_at) in different organs is plotted as reads per kilobase of transcript per million reads mapped (RPKM) using raw data acquired from ExpAt in *Lotus* Base^40^ (https://lotus.au.dk/expat/).

The lack of an effect on symbiotic nitrogen fixation in the *L. japonicus Ljahl1* and *Ljahl2* mutants could have different explanations. Possibly in *L. japonicus* (and it is imaginable that in *Medicago* or other legumes also) these AHLs do not induce genes required for nitrogen fixation but are essential for *NCR* gene regulation. Alternatively, in *L. japonicus* they may be functionally redundant in contrast to what we observed in *M. truncatula*. Such a difference could reflect the different patterns of nodule development in *L. japonicus* and *M. truncatula*. In *M. truncatula*, *MtAHL2* expression in roots decreased as nodules developed and conversely, low expression of *MtAHL1* in roots increased strongly during nodule development. This opens up the possibility that different ratios of AHL1 and AHL2 in trimeric complexes could differentially regulate *NCR169* expression at different stages during nodule development. Possibly in determinate *L. japonicus* nodules such complementary expression is not required. In soybean and *L. japonicus*, the expression of *AHL2* in both roots and nodules is higher than that of *AHL1* (Extended Data Fig. 9) and their pattern of expression would not appear to fit with differential expression during nodule development.

The identification of two new transcription factors required for development of symbiotic nitrogen fixation in legume nodules opens up a new phase in the analysis of the development of symbiotic nitrogen-fixing nodules. What other genes are regulated by MtAHL1 and MtAHL2 during nodule development? Is their essential role in nitrogen fixation limited to expression of *NCR* genes, as implied from the preliminary observation that mutation of each gene in *L. japonicus* did not block symbiotic nitrogen fixation but prevented *NCR169* expression? Do MtAHL1 and/or MtAHL2 regulate other genes in *M. truncatula* but not in *L. japonicus* nodules? Do MtAHLs form a hub where root- and/or nodule-specific transcription factors can repress and/or induce gene expression? Does MtAHL2 play some role in root development based on the observation that it is expressed in non-nodulated roots? Do other AHLs expressed in non-nodule tissues bind to the identified promoter element to suppress *NCR169* expression? Do these AHLs play a role during the development of determinate nodules with terminally differentiated bacteroids (that is, on *Aeschynomene*, *Arachis*) or indeterminate nodules with non-terminally differentiated bacteroids (for example, on *Leucaena*)? Answering these questions will shed light on the mechanisms governing the development of symbiotic nitrogen fixation in legumes.

## Methods

### Plant materials, hairy root transformation and nodulation assay

The plant materials were *M. truncatula* A17, soybean (*G. max* cv. Williams 82) and *L. japonicus* Gifu. Roots of these legumes were transformed using *Agrobacterium rhizogenes* strain ARqua-1 or K599 carrying specific vectors^28,29^. Nodules were induced on soybean by *B. japonicum* CB1809 when the *NCR169* promoter activity was investigated and by *B. japonicum* USDA110 wild-type, its *ΔbclA* mutant, when the effects of NCR169 on bacteroids were tested. *L. japonicus* was inoculated with *M. loti* R7A and *M. truncatula* by *S. medicae* WSM419. Plants for nodulation were grown in vermiculite and fertilized once per week with 1 g l^−1^ of Plant-Prod fertilizer (0–15–40, N–P–K; Brampton) in a growth chamber programmed for 16 h light and 8 h dark, at 28 °C/22 °C day/night for soybean and at 22 °C/20 °C day/night for *M. truncatula* and *L. japonicus*.

### Plasmid and vectors

The activity of the *NCR169* promoter was analysed using pCAMBIA3301 (CAMBIA) modified by replacement of the CaMV35S promoter upstream of the GUS reporter either with the 1,181 bp or 436 bp promoter regions from *NCR169*, or with the deleted derivative of the 436 bp fragment lacking nucleotides −157 bp to −121 bp. To assess the effect of *NCR169* on bacteroids in soybean nodules, the gene including the 1,181 bp promoter and all the exons and introns was first introduced into pENTR2B (Thermo Fisher Scientific) donor vector and then ligated into pKGW-RR-MGW^30^ destination vector via the LR-clonase reaction. For complementation of the *M. truncatula dnf7-2* mutant, the genomic fragment of *NCR169* was cloned into pCAMBIA2201 (CAMBIA) downstream of the 1,181 bp or 436 bp promoter regions, or the 436 bp region deleted for nucleotides −157 to −121. For generating the bait strain used in the Y1H assays, the 1,181 bp promoter region of *NCR169* was cloned into the XbaI and EcoRI sites of pHIS3NB^31,32^ and then cloned together with *HIS3* gene into the NotI and BamHI sites of pINT1 (refs. 31, 32) to make pINT1-*NCR169pr-HIS3*.

For CRISPR–Cas9 gene knockout of the *MtAHL* genes, oligonucleotides MtAHL1gRNA/MtAHL1gRNArc and MtAHL2gRNA/MtAHL2gRNArc were annealed and ligated into BsaI-digested pKSE401 (ref. 33). For RNA interference, cDNA fragments were cloned into pCR8/GW/TOPO and then recombined into the destination vector pUB-GWS-GFP^34^.

Proteins were expressed in *E. coli* strain BL21 carrying the constructs with the full-length gene coding sequences inserted into EcoRI/SalI sites of the pET28a(+) plasmid (Novagen).

The pUBC vector system^35^ was used for transient expression of proteins in *N. benthamiana* leaves. Gene coding regions were PCR amplified from cDNA and the products were ligated into pCR8/GW/TOPO via TOPO cloning (Invitrogen). The resulting donor clones were used in LR-mediated recombination into pUBC-GFP-DEST for analyses of cellular localization and pUBC-nYFP-DEST/pUBC-cYFP-DEST for bimolecular fluorescence complementation assays.

Primers used in creating the above constructs are shown in Supplementary Table 2.

### Protein purification and EMSA assays

Genes encoding His-tagged MtAHL1, MtAHL2 and GmAHL1 were generated by PCR using the primers shown in Supplementary Table 2, cloned into pET28a(+) and introduced into *E. coli* BL21. Overnight cultures (2 ml) grown at 37 °C were inoculated into 200 ml of LB medium and grown in a shaking incubator to OD_600_ = 0.5. Protein expression was then induced overnight at room temperature in shaken flasks by adding 0.05% l-arabinose and 0.25 mM isopropyl β-d-thiogalactoside. Bacteria were then pelleted by centrifugation (4,000*g*, 10 min, 4 °C), washed once with ice-cold water, resuspended in 4 ml of BS/THES buffer^36^ (22 mM Tris–HCl, pH 7.5, 4.4 mM EDTA, 8.9% (w/v) sucrose, 62 mM NaCl, 10 mM HEPES, 5 mM CaCl_2_, 50 mM KCl and 12% glycerol) supplied with 0.3% cOmplete protease inhibitor cocktail (Roche). The cells were disrupted on ice with cyclic sonication generated by a UIS250v ultrasonic processor (Hielscher Ultrasonics) with a 5 mm sonotrode (0.9 s sonication, 0.1 s pause, 90% amplitude (15 W) for 2 min) and then centrifuged at 16,000*g* at 4 °C for 2 min. The His-tagged proteins were purified from the supernatant using a column of HisPur cobalt resin (Thermo Fisher Scientific). Bound proteins were eluted with 1 ml of BS/THES buffer containing 150 mM imidazole, as described by the manufacturer. EMSA assays were conducted as described by Chen^37^, except that the bands were visualized by SYBR Gold staining. In the EMSA assay, a typical amount of 10 ng of probe and 200 ng of purified protein was used for one reaction. Titration EMSA was performed with fixed 10 ng of probe and purified protein in the range of 0, 25, 50, 100, 150, 200, 250 and 300 ng.

### DNA affinity pull-down assays

Nodule nuclei were isolated from *M. truncatula* and *G. max* by chopping nodules with a razorblade in prechilled nuclear isolation buffer (45 mM MgCl_2_, 30 mM trisodium citrate, 20 mM MOPS, 0.1% Triton X-100, pH 7.2–7.4). The suspensions were filtered first through a 100-μm pore size nylon mesh to remove cell debris. The nuclei going through a 30 μm filter were then collected by centrifugation at 1,500*g* for 10 min at 4 °C and resuspended in BS/THES buffer containing 0.3% cOmplete protease inhibitor cocktail. Nuclear proteins were released by vortexing for 5 s at every 3 min for 15 min.

For the DNA affinity pull-down experiment, 382 bp of *NCR169* promoter sequence (−386 bp to −4 bp) was amplified by two PCR reactions in 1.5 ml volume to generate probes with a biotin label on either end. PCR fragments were precipitated by adding 1/10 volume of 3 M sodium acetate and 1 volume of isopropanol at −80 °C overnight. Precipitated DNA was collected by centrifugation at 17,000*g* for 10 min and washed three times with 70% ethanol, then dissolved in nuclease-free H_2_O. The purity and concentration of the DNA fragments were checked by agarose gel electrophoresis and optical density measurements using Nanodrop.

DNA affinity pull-down assays used 200 μl of Dynabeads M280 (binding 40–80 μg of DNA) to bind the bait fragment pair essentially as described^36^ except that in the final step, the proteins bound to the beads were digested with trypsin to release peptides that were identified by MS analysis. The *M. truncatula* and *G. max* nodule proteins identified by MS analysis were compared using protein BLAST at the National Center for Biotechnology Information (http://blast.ncbi.nlm.nih.gov/) with the ‘align two sequences’ options and the default parameters.

### Y1H assays

The pINT1-*NCR169pr-HIS3* Y1H assay plasmid was linearized by EheI digestion and introduced by transformation^32^ into *S. cerevisiae* strain Y187 to generate the bait strain which was then transformed with plasmids generated from a *M. truncatula* EST library^17^. Transformants were plated on SD–Leu–His plates. Prey sequences were identified by sequencing and searches were done using BLAST on the Phytozome website (http://phytozome.jgi.doe.gov/) using the *M. truncatula* database.

### Transient protein expression in *N. benthamiana* leaves

*A. tumefaciens* AGL-1 strains transformed with the indicated constructs were grown and prepared for transient expression as described previously^38^. The cultures were resuspended at OD_600_ = 0.2 in infiltration buffer (10 mM MES pH 5.7, 10 mM MgCl_2_ and 100 mM acetosyringone). For co-expression, suspensions of different constructs were mixed in equal ratios and infiltrated into expanding leaves of 4-week-old *N. benthamiana* plants. The samples for microscopic imaging or GUS staining were collected 3 d after infiltration.

### Microscopy

Nodules after SYTO 13 or live/dead staining were observed by confocal microscopy as described^13^ using a Leica SP5 laser scanning confocal microscope (Leica). Transient green fluorescent protein (GFP) and yellow fluorescent protein (YFP) signals in *N. benthamiana* were observed by FluoView FV1000 (Olympus) confocal microscope.

### GUS staining

GUS staining of nodules and nodule sections was carried out as described^13^. Samples were fixed in ice-cold 90% acetone for 1 h, and then stained overnight at 37 °C with X-Gluc staining solution (containing 50 mM phosphate buffer pH 7.2, 0.5 mM K_3_Fe(CN)_6_ (potassium ferricyanide), 0.5 mM K_4_Fe(CN)_6_ (potassium ferro-cyanide) and 2 mM X-Gluc (Thermo Fisher Scientific)). *A. tumefaciens-*treated *N. benthamiana* leaves were collected 3 d after infection and were vacuum infiltrated for 15 min with X-Gluc staining solution containing 0.1% Triton X-100 and then incubated at 37 °C for 24 h. Chlorophyll was removed by washing the leaves with absolute ethanol before photography.

### Reporting summary

Further information on research design is available in the Nature Portfolio Reporting Summary linked to this article.

## Supplementary information

Reporting Summary

Supplementary Tables Supplementary Table 1. List of genes containing H0 motif (motif 4) in their 1 Kb promoter region based on FIMO analysis. Supplementary Table 2. List of primers used in this study.

## Data Availability

The data supporting the findings of this study are available within the paper and its supplementary information files. Source data (graphs) for Figs. 1–3 and Extended Data Figs. 1–9 are provided with this paper. Primers used in this study were listed in Supplementary Table 2. Proteins from the DNA affinity pull-down assay were identified and searched on UniProt (https://www.uniprot.org/). Genes identified from the Y1H screen were blasted and identified on Phytozome V13 (https://phytozome-next.jgi.doe.gov/).
