# Supplementary material for: Widely conserved AHL transcription factors are essential for NCR gene expression and nodule development in Medicago
Source: Nat Plants. 2023 Jan 9;9(2):280–8. doi: 10.1038/s41477-022-01326-4 (PMC9946822; doi:10.1038/s41477-022-01326-4)
Supplement: Supplementary file 1 — Reporting Summary [file 41477_2022_1326_MOESM1_ESM.pdf]

nature portfolio

Corresponding author(s): Eva Kondorosi

Last updated by author(s): Nov 29, 2022

Reporting Summary

Nature Portfolio wishes to improve the reproducibility of the work that we publish. This form provides structure for consistency and transparency in reporting. For further information on Nature Portfolio policies, see our Editorial Policies and the Editorial Policy Checklist.

Statistics

For all statistical analyses, confirm that the following items are present in the figure legend, table legend, main text, or Methods section.

n/a	Confirmed	
☐	☒	The exact sample size (*n*) for each experimental group/condition, given as a discrete number and unit of measurement
☒	☐	A statement on whether measurements were taken from distinct samples or whether the same sample was measured repeatedly
☐	☒	The statistical test(s) used AND whether they are one- or two-sided<br>*Only common tests should be described solely by name; describe more complex techniques in the Methods section.*
☒	☐	A description of all covariates tested
☒	☐	A description of any assumptions or corrections, such as tests of normality and adjustment for multiple comparisons
☐	☒	A full description of the statistical parameters including central tendency (e.g. means) or other basic estimates (e.g. regression coefficient) AND variation (e.g. standard deviation) or associated estimates of uncertainty (e.g. confidence intervals)
☐	☒	For null hypothesis testing, the test statistic (e.g. *F*, *t*, *r*) with confidence intervals, effect sizes, degrees of freedom and *P* value noted<br>*Give P values as exact values whenever suitable.*
☒	☐	For Bayesian analysis, information on the choice of priors and Markov chain Monte Carlo settings
☒	☐	For hierarchical and complex designs, identification of the appropriate level for tests and full reporting of outcomes
☒	☐	Estimates of effect sizes (e.g. Cohen's *d*, Pearson's *r*), indicating how they were calculated

Our web collection on statistics for biologists contains articles on many of the points above.

Software and code

Policy information about availability of computer code

Data collection	No software was used for data collection.
Data analysis	Flow cytometry data was analyzed on CytExpert 2.2.0.97. Confocal images for root nodule sections were analyzed with Leica TCS SP5 confocal laser scanning microscope (Germany). Confocal images for tobacco leaves were analyzed with Olympus FV1000 confocal microscope (Japan). Statistical analysis was performed with Microsoft Excel, or Graphpad Prism 5.

For manuscripts utilizing custom algorithms or software that are central to the research but not yet described in published literature, software must be made available to editors and reviewers. We strongly encourage code deposition in a community repository (e.g. GitHub). See the Nature Portfolio guidelines for submitting code & software for further information.

Data

Policy information about availability of data

All manuscripts must include a data availability statement. This statement should provide the following information, where applicable:

- Accession codes, unique identifiers, or web links for publicly available datasets
- A description of any restrictions on data availability
- For clinical datasets or third party data, please ensure that the statement adheres to our policy

The data supporting the findings of this study are available within the paper and its supplementary information files. Source Data (graphs) for Figs. 1-3 and Extended Data Fig. 1–9 are provided with the paper. Primers used in this study were listed in Supplementary Table S2. Proteins from DNA affinity pull-down assay were

identified and searched on UniProt (https://www.uniprot.org/). Genes identified from Y1H screen were blasted and identified on Phytozome V13 (https://phytozome-next.jgi.doe.gov/).

Human research participants

Policy information about studies involving human research participants and Sex and Gender in Research.

Reporting on sex and gender	n/a
Population characteristics	n/a
Recruitment	n/a
Ethics oversight	n/a

Note that full information on the approval of the study protocol must also be provided in the manuscript.

Field-specific reporting

Please select the one below that is the best fit for your research. If you are not sure, read the appropriate sections before making your selection.

☒ Life sciences ☐ Behavioural & social sciences ☐ Ecological, evolutionary & environmental sciences

For a reference copy of the document with all sections, see nature.com/documents/nr-reporting-summary-flat.pdf

Life sciences study design

All studies must disclose on these points even when the disclosure is negative.

Sample size	Sample size was determined based on previous publications on similar experiments to ensure confident statistical analyses. The sample size is indicated in the corresponding figure legends.
Data exclusions	No data were excluded from the analyses.
Replication	State number of times the experiment was replicated in laboratory. All experiments in this manuscript were independently replicated at least two times in the laboratory, and obtained the similar results.
Randomization	All samples were allocated randomly into experimental groups.
Blinding	The blinding design is not applicable to this system. Experiment results are not subjective.

Reporting for specific materials, systems and methods

We require information from authors about some types of materials, experimental systems and methods used in many studies. Here, indicate whether each material, system or method listed is relevant to your study. If you are not sure if a list item applies to your research, read the appropriate section before selecting a response.

Materials & experimental systems

n/a	Involved in the study	
☒	☐	Antibodies
☒	☐	Eukaryotic cell lines
☒	☐	Palaeontology and archaeology
☒	☐	Animals and other organisms
☒	☐	Clinical data
☒	☐	Dual use research of concern

Methods

n/a	Involved in the study	
☒	☐	ChIP-seq
☐	☒	Flow cytometry
☒	☐	MRI-based neuroimaging

Flow Cytometry

Plots

Confirm that:

- [x] The axis labels state the marker and fluorochrome used (e.g. CD4-FITC).
- [x] The axis scales are clearly visible. Include numbers along axes only for bottom left plot of group (a 'group' is an analysis of identical markers).
- [x] All plots are contour plots with outliers or pseudocolor plots.
- [x] A numerical value for number of cells or percentage (with statistics) is provided.

Methodology

Sample preparation	Root nodules were chopped with razor blades in PBS to release the bacteroids from the cells. Bacteroids was filtered to remove cell debris and stained with SYTO 13.
Instrument	CytoFLEX Flow Cytometer
Software	CytExpert
Cell population abundance	Each experiment was performed by recording 100, 000 events.
Gating strategy	Unstained debris was gated out by using unstained bacteroids from control and transgenic soybean root nodules.

- [x] Tick this box to confirm that a figure exemplifying the gating strategy is provided in the Supplementary Information.
